# Effects of sacubitril/valsartan on glycemia in patients with diabetes and heart failure: the PARAGON-HF and PARADIGM-HF trials

**DOI:** 10.1186/s12933-022-01545-1

**Published:** 2022-06-18

**Authors:** Magnus O. Wijkman, Brian Claggett, Muthiah Vaduganathan, Jonathan W. Cunningham, Rasmus Rørth, Alice Jackson, Milton Packer, Michael Zile, Jean Rouleau, Karl Swedberg, Martin Lefkowitz, Sanjiv J. Shah, Marc A. Pfeffer, John J. V. McMurray, Scott D. Solomon

**Affiliations:** 1 Cardiovascular Division, Brigham & Women’s Hospital, Harvard Medical School, 75 Francis Street, Boston, MA 02115 USA; 2 Department of Internal Medicine, Department of Health, Medicine and Caring Sciences, Linköping University, Norrköping, Sweden; 3 Rigshospitalet Copenhagen University Hospital, Copenhagen, Denmark; 4 British Heart Foundation Cardiovascular Research Centre, University of Glasgow, Glasgow, UK; 5 Baylor Heart and Vascular Institute, Baylor University Medical Center, Dallas, TX USA; 6 Imperial College, London, UK; 7 Medical University of South Carolina and Ralph H. Johnson Veterans Affairs Medical Center, Charleston, SC USA; 8 Montreal Heart Institute, University of Montreal, Montreal, Canada; 9 Department of Molecular and Clinical Medicine, University of Gothenburg, Gothenburg, Sweden; 10 Novartis, East Hanover, NJ USA; 11 Division of Cardiology, Department of Medicine, Northwestern University Feinberg School of Medicine, Chicago, IL USA

**Keywords:** Heart failure, Diabetes, Sacubitril/valsartan, Hypoglycemia

## Abstract

**Background:**

Compared with enalapril, sacubitril/valsartan lowered HbA1c and reduced new insulin therapy in patients with heart failure with reduced ejection fraction (HFrEF) and diabetes in the PARADIGM-HF trial. We sought to assess the glycemic effects of sacubitril/valsartan in heart failure with preserved ejection fraction (HFpEF) and diabetes, and across the spectrum of left ventricular ejection fraction (LVEF) in heart failure and diabetes.

**Methods:**

We compared the effect of sacubitril/valsartan, relative to valsartan, on HbA1c, new insulin therapy and hypoglycemia in the randomized controlled trial PARAGON-HF, and performed pooled analyses of PARAGON-HF and PARADIGM-HF.

**Results:**

Among 2395 patients with HFpEF and diabetes in PARAGON-HF, sacubitril/valsartan compared with valsartan reduced HbA1c (baseline-adjusted between-group difference in HbA1c change at 48 weeks: − 0.24%, 95% CI − 0.33 to − 0.16%, P < 0.001). Numerically, new insulin treatment was initiated less often in the sacubitril/valsartan group than in the valsartan group, but the difference was not statistically significant (12.8% vs. 16.1%; HR: 0.80, 95% CI 0.62–1.02, P = 0.07). Hypoglycemia adverse event reports were low, but more frequent in those receiving sacubitril/valsartan than in the valsartan group (4.2% vs. 2.6%; HR: 1.64, 95% CI 1.05–2.56, P = 0.030). In a pooled analysis of PARAGON-HF and PARADIGM-HF, the effect of sacubitril/valsartan on change in HbA1c was not significantly modified by LVEF (P_interaction_ = 0.56). Across the spectrum of LVEF, sacubitril/valsartan reduced new insulin therapy (HR: 0.75, 95% CI 0.63–0.89, P = 0.001), compared with enalapril or valsartan.

**Conclusions:**

Sacubitril/valsartan reduced HbA1c and new insulin therapy in patients with heart failure and diabetes across the spectrum of LVEF but may be associated with a slightly higher risk for hypoglycemia.

*Trial registration* ClinicalTrials.gov NCT01920711

**Supplementary Information:**

The online version contains supplementary material available at 10.1186/s12933-022-01545-1.

## Background

In patients with heart failure with preserved ejection fraction (HFpEF), diabetes is a common comorbidity which is associated with decreased survival and increased risk for heart failure hospitalization or cardiovascular death [1–5]. Poor glycemic control has been associated with increased risk for heart failure hospitalization [6, 7], and the requirement for insulin in particular has been associated with an increased risk for adverse cardiovascular outcomes in this population [5, 8, 9].

Treatment with the angiotensin receptor neprilysin inhibitor sacubitril/valsartan decreases morbidity and mortality in patients with heart failure with reduced ejection fraction (HFrEF) [10], and has also shown benefit in some patients with HFpEF [11]. Sacubitril/valsartan has recently received an expanded indication by the Food and Drug Administration (FDA) for treatment of chronic heart failure in a broader range of patients, with greatest benefits noted in patients with left ventricular ejection fraction (LVEF) below normal. Sacubitril/valsartan has previously been shown to reduce HbA1c levels and the need for insulin initiation in patients with HFrEF and diabetes [12] in the PARADIGM-HF trial. Furthermore, sacubitril/valsartan has been shown to improve measures of insulin sensitivity in obese patients who did not have diabetes or heart failure [13]. In the present study, we compared the effect of sacubitril/valsartan with that of valsartan on HbA1c, new use of antihyperglycemic drugs, and risk for hypoglycemia in patients with HFpEF and diabetes who participated in the PARAGON-HF trial. To better understand the impact of sacubitril/valsartan on glycemic control across the spectrum of LVEF, and to refine the estimates of the treatment effects in a larger sample, we also assessed these outcomes in a pooled analysis of patients from the PARAGON-HF and PARADIGM-HF trials.

## Methods

### Patients and study design

The PARAGON-HF trial (ClinicalTrials.gov NCT01920711) was a randomized, double-blind, active-controlled event-driven clinical trial that compared the efficacy and safety of sacubitril/valsartan with that of valsartan in 4796 patients with symptomatic HFpEF. As described in detail previously [14], patients were required to be at least 50 years old, to have LVEF of at least 45% within 6 months of screening, to have symptoms of heart failure corresponding to NYHA class II to IV, to have required diuretic therapy for at least 30 days before screening, and to have elevated levels of NT-proBNP and echocardiographic evidence of either left ventricular hypertrophy or left atrial enlargement or both. Patients were not eligible for inclusion if they had a prior LVEF of less than 40% or an alternative diagnosis that could explain their symptoms, but there were no inclusion or exclusion criteria related to diabetes, use of glucose lowering drugs, or glycemic control [14]. Patients who had tolerated two sequential single-blinded run-in periods (valsartan as monotherapy at half the target dose followed by sacubitril/valsartan at half the target dose) were randomized to double-blinded treatment with either sacubitril/valsartan 97/103 mg bid or valsartan 160 mg bid. The primary and secondary outcomes of the PARAGON-HF trial have been published previously [11]. The PARADIGM-HF trial was a randomized clinical trial that compared the efficacy and safety of sacubitril/valsartan with that of enalapril in 8399 patients with HFrEF [10, 15].

### Definitions of diabetes, glucose lowering medications and glycemic control

For this secondary analysis of the PARAGON-HF trial, patients were classified as having diabetes at baseline if at least one of the following three criteria were met: self-reported history of diabetes at screening, use of antihyperglycemic medications, or HbA1c of 6.5% or higher at randomization. The following medications were defined as antihyperglycemic drugs: insulin, biguanides, sulfonylureas, alpha-glucosidase inhibitors, thiazolidinediones, dipeptidyl peptidase-4 inhibitors, sodium-glucose cotransporter-2 (SGLT-2) inhibitors, glucagon-like peptide-1 (GLP-1) receptor agonists and glinides. Fixed combinations of oral drugs (used by 92 participants) were categorized into their component classes. Assessments of glycemic control (by HbA1c measurements) and of medication use were performed at the randomization visit and after 48, 96 and 144 weeks. HbA1c was measured by the BioRad D-10 ion-exchange high-performance liquid chromatography method [16]. During follow-up, the start dates for new use of antihyperglycemic drugs were recorded. New-onset diabetes was an adjudicated end-point in PARAGON-HF, which required at least one of the following criteria to be fulfilled: fasting plasma glucose ≥ 126 mg/dl on two occasions; HbA1c ≥ 6.5% on two occasions; fasting plasma glucose ≥ 126 mg/dl followed by HbA1c ≥ 6.5% (or vice versa); random non-fasting glucose ≥ 200 mg/dl and subsequent fasting plasma glucose ≥ 126 mg/dl; two-hour post load glucose ≥ 200 mg/dl after oral glucose tolerance test with an equivalent of 75 g of glucose and either a random (non-fasting) glucose ≥ 200 mg/dl on a different day or a fasting plasma glucose ≥ 126 mg/dl on a different day; confirmed use of diabetes drugs. Thus, the adjudicated new-onset diabetes events represented diabetes diagnosed in clinical practice outside of the study protocol as reported by the investigators and were not based on HbA1c measurements obtained as part of the study protocol. Hypoglycemia was not an adjudicated end-point, but was assessed as investigator-reported adverse events.

### Statistics

Continuous variables were summarized as mean ± standard deviation, or as median [IQR], and categorical variables were summarized using counts and percentages. Baseline between-group differences were tested for statistical significance with Student’s t-test, Wilcoxon’s rank-sum test or with the Chi-square test, as appropriate. Between-group differences in change in HbA1c from randomization to each of the follow-up measurements were assessed with linear regression models in which change in HbA1c was the outcome, treatment group was the predictor, and randomization HbA1c value was a covariate. Treatment-induced reductions in levels of circulating natriuretic peptides correlate with therapeutic benefits in terms of reduced risk for heart failure hospitalizations [17], and in both PARAGON-HF [18] and PARADIGM-HF [19], larger reductions of this biomarker were observed with sacubitril/valsartan, and were predictive of reduced morbidity and mortality rates. Therefore, to assess the possible contribution of improvements in heart failure severity to changes in HbA1c, we performed a separate linear regression model which adjusted additionally for simultaneous changes in levels of NT-proBNP. Overall differences between treatment groups regarding change in HbA1c during follow-up were tested for statistical significance with a longitudinal mixed model, with patient-level random intercept terms, fixed effects of treatment and time as a continuous variable, which assumed an unstructured covariance pattern. The impact of treatment on time to new use of antihyperglycemic medications, and on time to first hypoglycemic adverse events, was assessed with Cox proportional hazards models. Kaplan–Meier curves were used to visualize the cumulative incidence of new use of antihyperglycemic medications and of first hypoglycemic adverse events over time. Pooled analyses of the primary outcomes of the PARAGON-HF and PARADIGM-HF trials were pre-specified, and have been published previously [20]. In the present study, we pooled patient-level data of the two trials to assess potential interactions between LVEF and the treatment effects of sacubitril/valsartan on HbA1c change and on new use of antihyperglycemic drugs and on first hypoglycemic adverse events, and to calculate pooled estimates of the effect of treatment on these outcomes. The treatment effects of sacubitril/valsartan vs. control (estimated rate ratios and 95% confidence intervals obtained from negative binomial regression models) were estimated as a function of LVEF using restricted cubic splines with three knots. We considered two-sided P values < 0.05 as evidence for statistical significance. Statistical analyses were performed in STATA, version 14 (College Station, TX).

## Results

### Baseline characteristics

The baseline characteristics of the PARAGON-HF participants are presented by diabetes status in Additional file 1: Table S1. As shown in Additional file 1: Fig. S1, there were 2395 patients who were identified as having diabetes according to their medical history (n = 2062) and/or use of antihyperglycemic drugs (n = 1761) and/or having HbA1c ≥ 6.5% (n = 1816). Patients with diabetes were, on average, younger, had higher systolic blood pressure, and were more likely to be male, to be obese, to have had a myocardial infarction, and to have been hospitalized for heart failure. In patients with diabetes, baseline characteristics were similar between randomization groups (Table 1). Metformin was the most frequently used glucose-lowering medication (used by 1135 patients or 47.4% of the patients with diabetes) followed by insulin (used by 657 patients or 27.4% of the patients with diabetes) and sulfonylureas (used by 480 patients or 20.0% of the patients with diabetes). There were 634 patients with diabetes (312 in the valsartan group and 322 in the sacubitril/valsartan group, P = 0.90) who did not use any antihyperglycemic medications at the randomization visit.

**Table 1:** Baseline characteristics of PARAGON-HF participants with diabetes by randomization

	Valsartan, n = 1184	Sacubitril/valsartan, n = 1211	P
Demographics			
Age, years	72.0 ± 8.3	72.1 ± 8.3	0.61
Female, n (%)	588 (49.7%)	593 (49.0%)	0.73
Race/ethnicity, n (%)			0.63
Asian	153 (12.9%)	165 (13.6%)	
Black or African American	34 (2.9%)	27 (2.2%)	
Other	42 (3.5%)	50 (4.1%)	
White	955 (80.7%)	969 (80.0%)	
Enrollment region, n (%)			0.84
Asia/Pacific	192 (16.2%)	209 (17.3%)	
Central Europe	444 (37.5%)	444 (36.7%)	
Latin America	75 (6.3%)	83 (6.9%)	
North America	146 (12.3%)	158 (13.0%)	
Western Europe	327 (27.6%)	317 (26.2%)	
Comorbidities, n (%)			
Prior MI	306 (25.8%)	333 (27.5%)	0.36
Ischemic etiology	476 (40.2%)	507 (41.9%)	0.40
Atrial fibrillation	369 (31.4%)	401 (33.1%)	0.36
Prior HF hospitalization	631 (53.3%)	622 (51.4%)	0.34
Hypertension	1145 (96.7%)	1170 (96.6%)	0.90
Stroke	120 (10.2%)	152 (12.6%)	0.06
Obesity (BMI ≥ 30 kg/m^2^)	686 (57.9%)	679 (56.1%)	0.36
CKD (eGFR < 60 ml/min/1.73 m^2^)	614 (51.9%)	593 (49.0%)	0.16
NYHA functional class, n (%)			0.51
1	42 (3.5%)	37 (3.1%)	
2	890 (75.2%)	910 (75.2%)	
3	245 (20.7%)	260 (21.5%)	
4	7 (0.6%)	3 (0.2%)	
LVEF (percent)	57.2 ± 8.0	57.2 ± 7.8	0.98
SBP, mmHg	131 ± 15	132 ± 16	0.45
DBP, mmHg	74 ± 10	74 ± 11	0.79
Heart rate, bpm	71 ± 12	72 ± 12	0.07
BMI, kg/m^2^	31.2 ± 4.9	30.9 ± 4.9	0.16
eGFR, ml/min/1.73 m^2^	62 ± 20	63 ± 19	0.29
HbA1c, percent	7.32 ± 1.43	7.37 ± 1.48	0.44
NT-proBNP, pg/ml	578 [281–1125]	564 [282–1142]	0.66
Medications, n (%)			
Beta blockers	969 (81.8%)	998 (82.4%)	0.72
Diuretics	1138 (96.1%)	1159 (95.7%)	0.61
MRA	316 (26.7%)	308 (25.4%)	0.48
Insulin	319 (26.9%)	338 (27.9%)	0.60
GLP-1 receptor agonists	10 (0.8%)	10 (0.8%)	0.96
Oral glucose lowering			
Metformin	563 (47.6%)	572 (47.2%)	0.88
Sulfonylurea	250 (21.1%)	230 (19.0%)	0.19
SGLT-2 inhibitors	14 (1.2%)	14 (1.2%)	0.95
DPP-4 inhibitors	150 (12.7%)	148 (12.2%)	0.74
Alpha glucosidase inhibitors	40 (3.4%)	50 (4.1%)	0.33
Thiazolidinediones	14 (1.2%)	8 (0.7%)	0.18
Others (glinides)	25 (2.1%)	31 (2.6%)	0.47

Data are presented as mean ± SD, median [Q1–Q3] or n (%)

*BMI* body mass index, *CCB* calcium channel blocker, *CKD* chronic kidney disease, *DBP* diastolic blood pressure, *DPP-4* dipeptidyl peptidase-4, *eGFR* estimated glomerular filtration rate, *GLP-1* glucagon-like peptide-1, *HF* heart failure, *LVEF* left ventricular ejection fraction, *MI* myocardial infarction, *MRA* mineralocorticoid receptor antagonists, *NT-proBNP* N-terminal prohormone B-type natriuretic peptide, *SBP* systolic blood pressure, *SGLT-2* sodium glucose cotransporter-2

### Changes in HbA1c

**Fig. 1:**
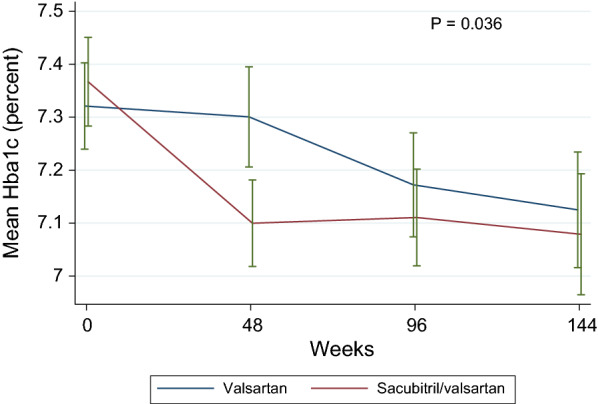
Mean HbA1c values at randomization and at follow-up, in PARAGON-HF participants with diabetes, by treatment groups. Error bars represent 95% confidence intervals. P from mixed model for equality of HbA1c slopes

At randomization, HbA1c values were available in all but one patient with diabetes, and there was no significant difference in mean HbA1c values between the sacubitril/valsartan and the valsartan groups (7.37 ± 1.48% vs. 7.32 ± 1.43%, P = 0.44). Mean values of HbA1c at randomization and at weeks 48, 96 and 144 are shown in Fig. 1 and in Table 2 by randomization groups. Overall, there was a significantly larger reduction in HbA1c in the sacubitril/valsartan group than in the valsartan group (P from mixed model for equality of HbA1c slopes = 0.036). The baseline-adjusted between-group difference in changes in mean HbA1c values was largest at week 48 (− 0.24%, 95% CI − 0.33 to − 0.16%, P < 0.001). In patients who also had available data for NT-proBNP at randomization and at week 48 (n = 1489), this difference remained significant after additional adjustment for change in NT-proBNP (− 0.20, 95% CI − 0.31 to − 0.10, P < 0.001). Baseline BMI did not significantly modify the treatment effect of sacubitril/valsartan compared with valsartan on HbA1c (P_interaction_ = 0.44). There was no significant association between change in HbA1c and change in eGFR (P = 0.64). The difference between treatment-groups was attenuated over time but remained statistically significant also at week 96 and 144 (Table 2). In the 2184 patients without diabetes at baseline and with HbA1c values measured both at randomization and at week 48, sacubitril/valsartan (n = 1094) was associated with a significantly larger reduction in HbA1c than valsartan (n = 1090), but of a considerably smaller magnitude (baseline-adjusted between-group difference: − 0.04%, 95% CI − 0.04 to − 0.01%, P = 0.013) than among the patients with diabetes at baseline.

**Table 2:** Mean HbA1c values at randomization and at follow-up, in PARAGON-HF participants with diabetes, by treatment groups

	Valsartan, n = 1184	Sacubitril/valsartan, n = 1211	Adjusted difference in change from randomization (95% CI)	P value
HbA1c (percent)				
Randomization	7.32 ± 1.43	7.37 ± 1.48	–	–
Week 48	7.30 ± 1.57	7.10 ± 1.37	− 0.24 (− 0.33 to − 0.16)	< 0.001
Week 96	7.17 ± 1.55	7.11 ± 1.46	− 0.12 (− 0.22 to − 0.01)	0.027
Week 144	7.13 ± 1.46	7.08 ± 1.52	− 0.13 (− 0.26 to − 0.003)	0.044

Between-group differences in changes from randomization were adjusted for randomization HbA1c values. Number of patients with HbA1c measurements at randomization: n = 2394 (val: n = 1183, sac/val: n = 1211), at week 48: n = 2143 (val: n = 1056, sac/val: n = 1087), at week 96: n = 1947 (val: n = 958, sac/val: n = 989), at week 144: n = 1378 (val: n = 693, sac/val: n = 685)

### New use of antihyperglycemic medications

At randomization, there were 1738 patients with diabetes who did not use insulin. The proportion of patients with diabetes who initiated insulin treatment during follow-up was lower in the sacubitril/valsartan group than in the valsartan group, but the difference did not reach statistical significance (12.8% vs. 16.1%; HR: 0.80, 95% CI 0.62–1.02, P = 0.07). Among the 634 patients with diabetes who did not use any antihyperglycemic drugs at randomization, the proportions of patients who initiated treatment with non-insulin antihyperglycemic medications were similar between treatment groups (13.7% vs. 15.7%; HR: 0.88, 95% CI 0.58–1.32, P = 0.53). The proportions of patients who initiated treatment with metformin did also not differ significantly between groups (9.3% vs. 13.5%, HR 0.69, 95% CI 0.43–1.09, P = 0.11).

**Fig. 2:**
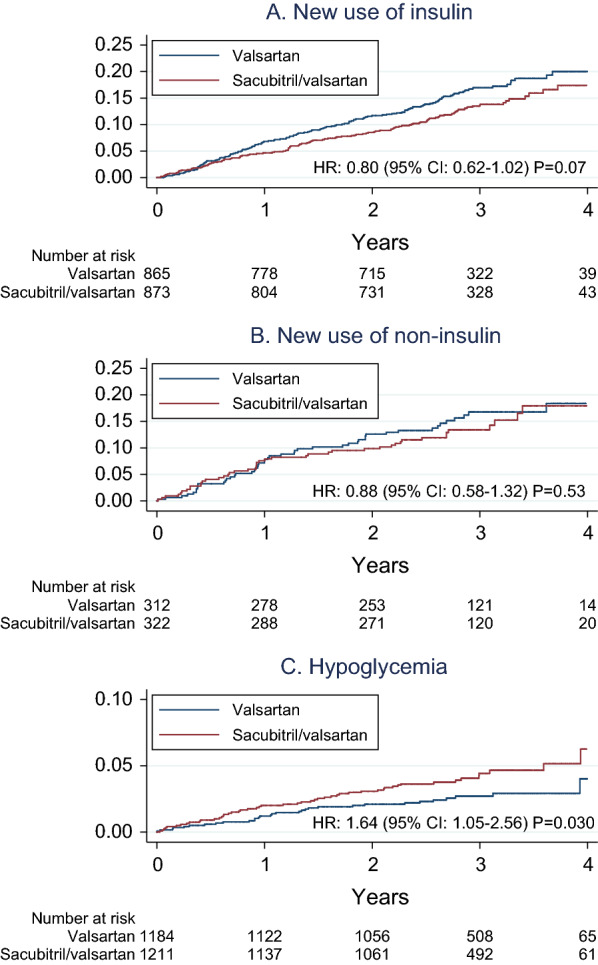
Cumulative incidence of different outcomes in PARAGON-HF participants with diabetes. **A** New use of insulin. **B** New use of non-insulin antihyperglycemic medications. **C** Hypoglycemia. *HR* hazard ratio, *CI* confidence interval

The proportion of women was similar in patients who initiated or not initiated insulin treatment (46.6% vs. 50.4%, P = 0.27) and in patients who initiated or not initiated non-insulin antihyperglycemic medications (47.3% vs. 54.3%, P = 0.21), respectively. The cumulative incidences of new use of insulin and of non-insulin antihyperglycemic medications, respectively, are shown by treatment groups in Fig. 2.

### Hypoglycemia

Overall, 82 patients with diabetes had at least one investigator-reported hypoglycemic adverse event. Sixty-seven of these events (81.7%) occurred in patients who used either insulin or sulfonylurea (or both) at baseline. The distribution of first hypoglycemic adverse events is shown in Additional file 1: Table S2. Patients with diabetes who experienced at least one hypoglycemic adverse event were of similar age as patients with diabetes who did not experience any hypoglycemic adverse event (72.3 ± 7.9 years vs. 72.0 ± 8.3 years, P = 0.78), and there was no significant difference in the proportion of women (50.0% vs. 49.3%, P = 0.90). Patients with diabetes who experienced at least one hypoglycemic adverse event had significantly higher baseline HbA1c (7.67 ± 1.50% vs. 7.33 ± 1.46%, P = 0.042), were more likely to use insulin (62.2% vs. 26.2%, P < 0.001) or sulfonylureas (28.0% vs. 19.8%, P = 0.07) but the latter finding did not meet statistical significance. The incidence of first hypoglycemic adverse events was significantly higher in patients with diabetes in the sacubitril/valsartan group than in patients with diabetes in the valsartan group (4.2% vs. 2.6%; HR 1.64, 95% CI 1.05–2.56, P = 0.030). In the 1064 patients with diabetes who used either insulin or sulfonylurea or both at baseline, the same conclusion was drawn but the incidence of first hypoglycemic adverse events was higher (7.8% vs. 4.8%; HR 1.66, 95% CI 1.02–2.71, P = 0.043), whereas in patients with diabetes who used neither insulin nor sulfonylurea at baseline (n = 1331), no significantly increased risk was observed in the sacubitril/valsartan group (1.5% vs. 0.8%; HR 1.92, 95% CI 0.65–5.61, P = 0.24).

In patients with diabetes who used metformin but who did not use neither insulin nor sulfonylurea at baseline (n = 593), there were only nine first hypoglycemic adverse events and there was not a significantly higher risk for hypoglycemic adverse events in the sacubitril/valsartan group (2.0% vs. 1.0%; HR 1.92, 95% CI 0.48–7.69, P = 0.36). In an analysis of all patients (with or without diabetes) who did not use glucose-lowering medications at baseline (n = 3035), there were 7 first hypoglycemic adverse events and no significantly increased risk was observed in the sacubitril/valsartan group (0.3% vs. 0.2%; HR 1.31, 95% CI 0.29–5.86, P = 0.72). The cumulative incidence of first hypoglycemic adverse events in patients with diabetes is shown by randomization groups in Fig. 2.

### Body weight, triglycerides, and new diabetes

Mean values of body weight and of triglyceride levels at randomization and at weeks 48, 96 and 144 are shown in Additional file 1: Fig. S2, Table S3. In patients with diabetes, body weight increased more in patients randomized to sacubitril/valsartan than to valsartan (average yearly treatment effect of sacubitril/valsartan: 0.27 kg (95% CI 0.06 to 0.49 kg, P = 0.012) and triglycerides increased more in patients randomized to valsartan (average yearly treatment effect of sacubitril/valsartan: − 0.05 mmol/l (95% CI − 0.08 to − 0.02 mmol/l, P = 0.003). Among the 2401 patients who did not have diabetes at randomization, the incidence of adjudicated new-onset diabetes was 9/1196 in the sacubitril/valsartan group and 12/1205 in the valsartan group (HR 0.74, 95% CI 0.31–1.76, P = 0.50).

### Pooled analyses of PARAGON-HF and PARADIGM-HF

**Fig. 3:**
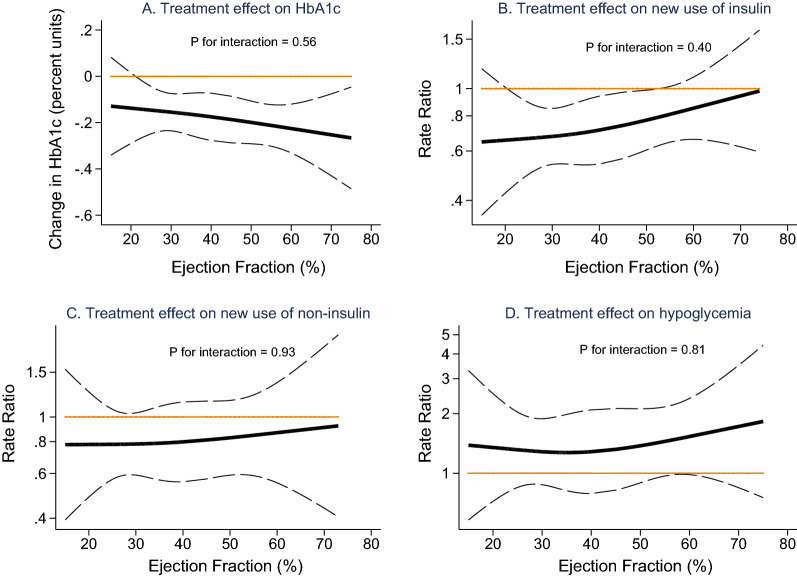
Treatment effects of sacubitril/valsartan compared with control treatment (valsartan in PARAGON-HF and enalapril in PARADIGM-HF) in patients with heart failure and diabetes, by left ventricular ejection fraction. **A** HbA1c reduction from randomization to 48 weeks (n = 5241). **B** New use of insulin (n = 4778). **C** New use of non-insulin antihyperglycemic medications (n = 2232). **D** Hypoglycemia (n = 6173)

To assess the effects on glycemia of sacubitril/valsartan across the spectrum of LVEF in patients with heart failure, we explored interactions between LVEF and treatment effects in a pooled patient-level analysis of patients with diabetes from the PARAGON-HF and PARADIGM-HF trials. Pooled analyses of the primary outcomes of these two heart failure trials were pre-specified, and have been published previously [20]. Left ventricular ejection fraction did not significantly modify the treatment effect of sacubitril/valsartan compared with control treatment on HbA1c (P_interaction_ = 0.56, Fig. 3), and neither did baseline BMI (P_interaction_ = 0.06). There was no significant association between change in HbA1c and change in eGFR (P = 0.08).

At 48 weeks, the reduction in mean HbA1c was larger in the sacubitril/valsartan group than in the control group, with a baseline-adjusted between-group difference in change in mean HbA1c of − 0.19% (95% CI − 0.25 to − 0.13%, P < 0.001). In patients who also had available data for NT-proBNP at randomization and at week 48 (PARAGON-HF, n = 1489) or week 32 (PARADIGM-HF, n = 757), this difference remained significant after additional adjustment for change in NT-proBNP (− 0.17, 95% CI − 0.25 to − 0.09, P < 0.001). The proportions of patients with diabetes who initiated insulin treatment during follow-up was significantly lower in the sacubitril/valsartan group than in the control group (9.3% vs. 12.4%; HR 0.75, 95% CI 0.63–0.89, P = 0.001), and this was not significantly modified by LVEF (P_interaction_ = 0.40; Fig. 3). Among the patients with diabetes who did not use any antihyperglycemic medications at randomization, the proportion who initiated treatment with non-insulin antihyperglycemic medications was numerically lower in the sacubitril/valsartan group than in the control group (11.5% vs. 14.3%; HR 0.80, 95% CI 0.64–1.01, P = 0.06) and this was not significantly modified by LVEF (P_interaction_ = 0.93; Fig. 3). New use of metformin also occurred less often in the sacubitril/valsartan group than in the control group (6.8% vs. 9.2%, HR 0.73, 95% CI 0.54–0.98, P = 0.035).

Overall, there were 179 patients with diabetes who had at least one investigator-reported hypoglycemic adverse event. One hundred and forty-nine of these events (83.2%) occurred in patients who used either insulin or sulfonylurea (or both) at baseline. The distribution of first hypoglycemic adverse events is shown in Additional file 1: Table S2. The incidence of first hypoglycemic adverse events was significantly higher in patients with diabetes randomized to sacubitril/valsartan than in patients with diabetes randomized to the control group (3.3% vs. 2.5%; HR: 1.37, 95% CI 1.02–1.84, P = 0.038) and without a significant interaction between LVEF and treatment effect (P_interaction _= 0.81, Fig. 3). In the subgroup of patients with diabetes who used either insulin or sulfonylurea or both at baseline (n = 2493) the risk was higher with sacubitril/valsartan (7.0% vs. 5.0%; HR 1.42 (95% CI 1.03–1.97, P = 0.034), whereas in patients with diabetes who used neither insulin nor sulfonylurea at baseline (n = 3680) no significantly increased risk was observed in the sacubitril/valsartan group (1.0% vs. 0.7%; HR 1.43, 95% CI 0.69–2.97, P = 0.34). In patients with diabetes who used metformin but who did not use neither insulin nor sulfonylurea at baseline (n = 1035) there were 16 first hypoglycemic adverse events and there was not a significantly higher risk for hypoglycemic adverse events in the sacubitril/valsartan group (1.9% vs. 1.2%; HR 1.57, 95% CI 0.57–4.31, P = 0.39). In an analysis of all patients (with or without diabetes) who did not use glucose-lowering medications at baseline (n = 9261), there were 17 first hypoglycemic adverse events and no significantly increased risk was observed in the sacubitril/valsartan group (0.2% vs. 0.1%; HR 1.81, 95% CI 0.67–4.90, P = 0.24). The cumulative incidences of new use of insulin, of new use of non-insulin antihyperglycemic medications, and of first hypoglycemic adverse events in the pooled cohort are shown by randomization groups in Additional file 1: Fig. S3.

## Discussion

We found that in patients with HFpEF and diabetes, sacubitril/valsartan significantly reduced HbA1c compared with valsartan, and that new use of insulin was numerically lower in patients randomized to sacubitril/valsartan than in patients randomized to valsartan. In a pooled analysis of patient-level data from patients in the PARADIGM-HF and PARAGON-HF trials, the effects of treatment with sacubitril/valsartan on HbA1c reduction and on new use of insulin were similar across the spectrum of LVEF. While these results suggest potentially beneficial effects, we also found a slightly increased risk for hypoglycemic adverse events with sacubitril/valsartan. These findings have implications for the treatment of patients with heart failure and diabetes.

Our finding that sacubitril/valsartan reduced HbA1c in patients with HFpEF and diabetes are consistent with what was observed in patients with HRrEF and diabetes in the PARADIGM-HF trial [12], in which sacubitril/valsartan reduced HbA1c compared with enalapril. The incremental reductions in HbA1c observed with sacubitril/valsartan compared with valsartan in PARAGON-HF or with enalapril in PARADIGM-HF are small, but appear to be consistent across the spectrum of LVEF and to be sufficient to decrease the requirement for insulin. Expectedly, the reduction of HbA1c that was observed with sacubitril/valsartan in patients without diabetes at baseline was considerably smaller in magnitude, which may be an explanation to why we did not observe a reduced incidence of new diabetes. We did not observe any significant associations between changes in HbA1c and changes in renal function, which may be due to the small magnitude of the HbA1c reduction and the relatively short follow-up time.

The mechanisms by which sacubitril/valsartan might lower HbA1c remain unclear. Sacubitril inhibits the endopeptidase neprilysin which promotes the proteolytic cleavage and inactivation of natriuretic peptides and other vasoactive peptides [21]. In observational cohort studies, higher levels of natriuretic peptides have been associated with lower measures of insulin resistance in obese as well as non-obese individuals [22], and with decreased risk for new-onset diabetes [23, 24]. Mendelian randomization studies of a common variant of the B-type natriuretic peptide (BNP) gene locus have suggested that these inverse relationships between natriuretic peptide levels and the risk for new onset diabetes may be causal [25]. The mechanisms by which natriuretic peptides may affect glucose metabolism and insulin resistance remain to be determined, but may involve increased secretion of the insulin-sensitizing hormone adiponectin, decreased secretion of pro-inflammatory cytokines from the adipose tissue, and increased insulin secretion [26]. An alternative explanation for the beneficial glycemic effects observed with sacubitril/valsartan may be decreased degradation of the antihyperglycemic incretin hormone glucagon-like peptide-1 (GLP-1) [27]. Glucagon-like peptide-1 is a known substrate for neprilysin in vitro [28], and animal studies have suggested that up to 50% of exogenously infused GLP-1 may be degraded by neprilysin [29]. In patients with heart failure who were switched from angiotensin converting enzyme inhibitors or angiotensin receptor blockers to sacubitril/valsartan, a dose-dependent increase of circulating GLP-1 levels has been described, which correlated with a decrease in levels of the protein glycation biomarker fructosamine [30]. Finally, it is known that insulin resistance increases with increasing heart failure severity [31], and although the treatment effect on HbA1c remained statistically significant after adjustment for changes in levels of NT-proBNP, it remains plausible that sacubitril/valsartan improved measures of glycemia by reducing worsening of heart failure progression over time.

Observational data have shown that use of insulin is associated with an adverse prognosis in people with heart failure and diabetes [5, 8, 9], and that higher HbA1c is a risk factor for developing heart failure in people with diabetes [6, 7]. Therefore, although the treatment effects were numerically small, the improvement in HbA1c and the reduced need to initiate insulin treatment that was observed with sacubitril/valsartan in this secondary analysis may benefit people with heart failure and diabetes. The slightly increased risk for hypoglycemia that was observed should not discourage use of sacubitril/valsartan, given its beneficial effects on morbidity and mortality [10, 11]. It should also be noted that in stratified analyses, sacubitril/valsartan was associated with significantly higher risk for hypoglycemic adverse events only in patients with diabetes who used either insulin or sulfonylurea at baseline. In people with heart failure and diabetes who experience hypoglycemia, conventional strategies should be recommended (such as patient education, increased frequency of self-measurement of blood glucose, and possibly dose adjustments of insulin or sulfonylurea) regardless of whether the patient is treated with sacubitril/valsartan.

### Study limitations and strengths

Some limitations of this analysis should be noted. The PARAGON-HF trial was not primarily designed to detect changes in HbA1c, changes in antihyperglycemic medications, or hypoglycemia. Changes in HbA1c were observed against the background of therapeutic treatment decisions that took place outside of the study protocol. Hypoglycemia was assessed as investigator-reported adverse events without a requirement for biochemical confirmation. While new onset diabetes was a pre-specified exploratory outcome in PARAGON-HF, there were a very small number of adjudicated cases of new diabetes, and although the adjudication criteria required hyperglycemia to be present on at least two occasions, the adjudication criteria did not specifically mandate confirmation of hyperglycemia with two different test methods. The statistical power for this outcome was low. We did not have data that allowed us to differentiate between type 1 and type 2 diabetes. Finally, we did not analyze dose adjustments or changes between different drug classes of antihyperglycemic medications. Strengths of this study include the randomized controlled design and the careful characterization of all patients at baseline and during follow-up. The pooled analyses of patients from the PARAGON-HF and PARADIGM-HF trials made exploratory analyses across the spectrum of LVEF possible, including patients who fulfilled the recently proposed criteria [32] for either HFrEF, heart failure with mildly reduced ejection fraction (HFmrEF) or HFpEF.

## Conclusions

In summary, these data indicate that the glucose-lowering effect of sacubitril/valsartan, as evidenced by a small but significant reduction in HbA1c and new use of insulin, was similar in heart failure patients with diabetes across the spectrum of LVEF. While the overall magnitude of the HbA1c lowering effect of sacubitril/valsartan was modest and the overall occurrence of hypoglycemic adverse events was low, clinicians should be aware of the possibility of improved glycemic control and reduced need for insulin, when initiating therapy with sacubitril/valsartan in patients with diabetes.

## Supplementary Information

**Additional file 1: Figure S1.** Numbers of PARAGON-HF participants fulfilling the three criteria used to define diabetes at baseline. **Table S1.** Baseline characteristics of PARAGON-HF participants by diabetes status. **Table S2. **Distribution of first investigator-reported hypoglycemic adverse events (AE) in PARAGON-HF and PARADIGM-HF participants with diabetes. **Table S3.** Average body weight and triglyceride levels at randomization and at follow-up in PARAGON-HF participants with diabetes, by treatment groups. **Figure S2.** Average body weight and triglyceride levels at randomization and at follow-up, in PARAGON-HF participants with diabetes, by treatment groups. **Figure S3.** Cumulative incidence of different outcomes in PARAGON-HF and PARADIGM-HF participants with diabetes.

## Data Availability

The trial data availability is according to the criteria and process described on www.clinicalstudydatarequest.com.

## Abbreviations

BNP: B-type natriuretic peptide
FDA: Food and Drug Administration
GLP-1: Glucagon-like peptide-1
HFmrEF: Heart failure with mildly reduced ejection fraction
HFpEF: Heart failure with preserved ejection fraction
HFrEF: Heart failure with reduced ejection fraction
LVEF: Left ventricular ejection fraction
MRA: Mineralocorticoid receptor antagonist
PARADIGM-HF: Prospective comparison of angiotensin receptor–neprilysin inhibitor with angiotensin-converting-enzyme inhibitor to determine impact on global mortality and morbidity in heart failure
PARAGON-HF: The prospective comparison of angiotensin receptor–neprilysin inhibitor with angiotensin-receptor blockers global outcomes in hf with preserved ejection fraction
SGLT-2: Sodium–glucose cotransporter-2
