# Supplementary material for: Effects of sacubitril/valsartan on glycemia in patients with diabetes and heart failure: the PARAGON-HF and PARADIGM-HF trials
Source: Cardiovasc Diabetol. 2022 Jun 18;21:110. doi: 10.1186/s12933-022-01545-1 (PMC9206286; doi:10.1186/s12933-022-01545-1)
Supplement: Supplementary file 1 — Additional file 1: Figure S1. Numbers of PARAGON-HF participants fulfilling the three criteria used to define diabetes at baseline. Table S1. Baseline characteristics of PARAGON-HF participants by diabetes status. Table S2. Distribution of first investigator-reported hypoglycemic adverse events (AE) in PARAGON-HF and PARADIGM-HF participants with diabetes. Table S3. Average body weight and triglyceride levels at randomization and at follow-up in PARAGON-HF participants with diabetes, by treatment groups. Figure S2. Average body weight and triglyceride levels at randomization and at follow-up, in PARAGON-HF participants with diabetes, by treatment groups. Figure S3. Cumulative incidence of different outcomes in PARAGON-HF and PARADIGM-HF participants with diabetes. [file 12933_2022_1545_MOESM1_ESM.docx]

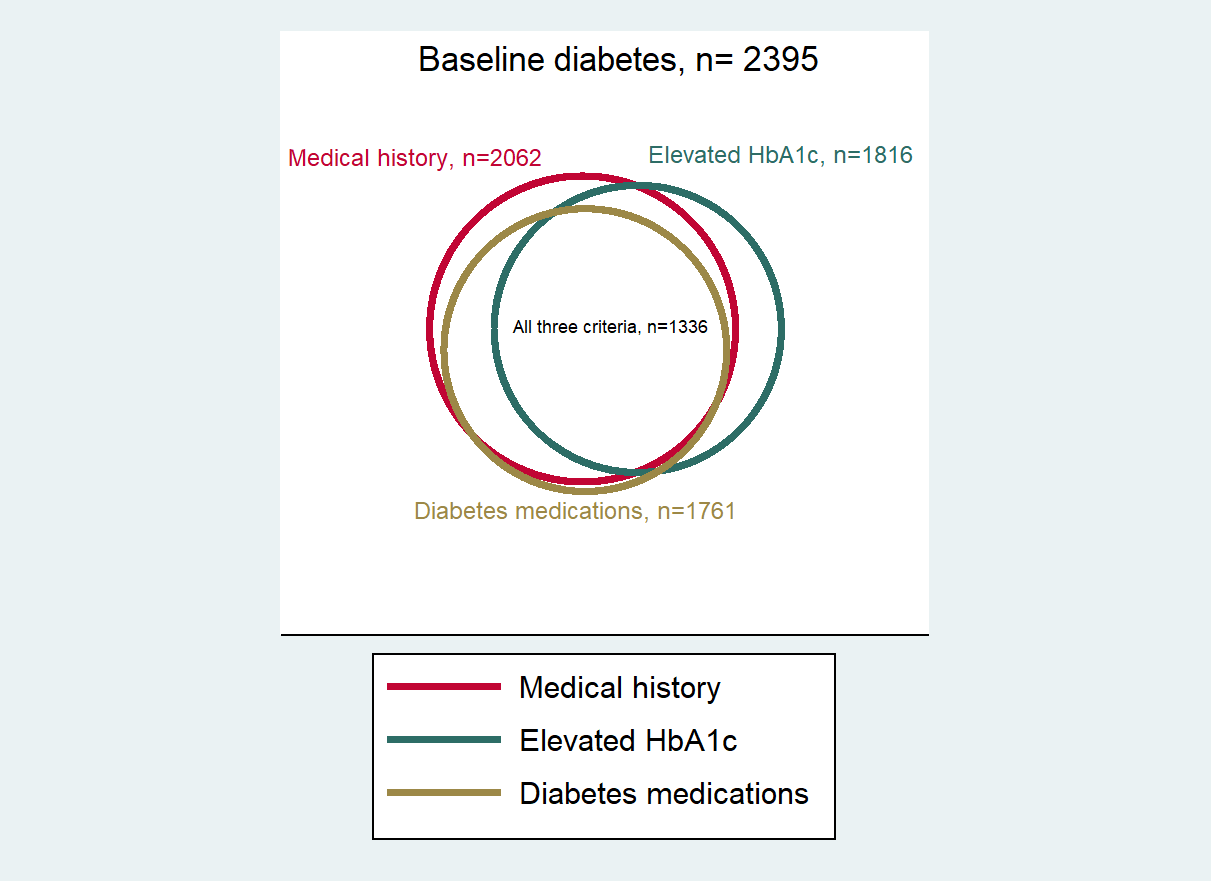


**Figure S1.** Numbers of PARAGON-HF participants fulfilling the three criteria used to define diabetes at baseline.

|  | No diabetes, n=2401 | Diabetes, n=2395 | P |
| --- | --- | --- | --- |
| Demographics |  |  |  |
| Age, years | 73.4 ± 8.5 | 72.0 ± 8.3 | <0.001 |
| Female, n (%) | 1298 (54.1%) | 1181 (49.3%) | <0.001 |
| Race/ethnicity, n (%) |  |  | 0.10 |
| Asian | 289 (12.0%) | 318 (13.3%) |  |
| Black or African American | 41 (1.7%) | 61 (2.5 %) |  |
| Other | 88 (3.7%) | 92 (3.8 %) |  |
| White | 1983 (82.6%) | 1924 (80.3%) |  |
| Enrollment region, n (%) |  |  | <0.001 |
| Asia/Pacific | 361 (15.0%) | 401 (16.7%) |  |
| Central Europe | 827 (34.4%) | 888 (37.1%) |  |
| Latin America | 212 (8.8%) | 158 (6.6%) |  |
| North America | 255 (10.6%) | 304 (12.7%) |  |
| Western Europe | 746 (31.1%) | 644 (26.9%) |  |
| Comorbidities, n (%) |  |  |  |
| Prior MI | 444 (18.5%) | 639 (26.7%) | <0.001 |
| Ischemic etiology | 740 (30.8%) | 983 (41.1%) | <0.001 |
| Atrial fibrillation | 782 (32.7%) | 770 (32.3%) | 0.79 |
| Prior HF hospitalization | 1053 (43.9%) | 1253 (52.3%) | <0.001 |
| Hypertension | 2269 (94.5%) | 2315 (96.7%) | <0.001 |
| Stroke | 236 (9.9%) | 272 (11.4%) | 0.09 |
| Obesity (BMI ≥30 kg/m^2^) | 992 (41.3%) | 1365 (57.0%) | <0.001 |
| CKD (eGFR <60 ml/min/1.73m^2^) | 1134 (47.2%) | 1207 (50.4%) | 0.028 |
| NYHA functional class, n (%) |  |  | 0.005 |
| 1 | 58 (2.4 %) | 79 (3.3 %) |  |
| 2 | 1906 (79.4%) | 1800 (75.2%) |  |
| 3 | 427 (17.8%) | 505 (21.1%) |  |
| 4 | 9 (0.4 %) | 10 (0.4 %) |  |
| LVEF (percent) | 57.8 ± 7.9 | 57.2 ± 7.9 | 0.006 |
| SBP, mmHg | 130 ± 15 | 132 ± 16 | <0.001 |
| DBP, mmHg | 74 ± 11 | 74 ± 10 | 0.56 |
| Heart rate, bpm | 70 ± 12 | 71 ± 12 | <0.001 |
| BMI, kg/m^2^ | 29.4 ± 5.0 | 31.1 ± 4.9 | <0.001 |
| eGFR, ml/min/1.73m^2^ | 63 ± 18 | 62 ± 20 | 0.19 |
| HbA1c, percent | 5.80 ± 0.36 | 7.34 ± 1.46 | NA |
| NT-proBNP, pg/ml | 545 [266, 1150] | 574 [282, 1138] | 0.31 |
| Medications, n (%) |  |  |  |
| Beta blockers | 1854 (77.2%) | 1967 (82.1%) | <0.001 |
| Diuretics | 2288 (95.3%) | 2297 (95.9%) | 0.30 |
| MRA | 615 (25.6%) | 624 (26.1%) | 0.73 |
| Insulin | - | 657 (27.4%) | NA |
| GLP-1 receptor agonists | - | 20 (0.8 %) | NA |
| Oral glucose lowering | - | 1482 (61.9%) | NA |
| Metformin | - | 1135 (47.4%) | NA |
| Sulfonylurea | - | 480 (20.0%) | NA |
| SGLT-2 inhibitors | - | 28 (1.2 %) | NA |
| DPP-4 inhibitors | - | 298 (12.4 %) | NA |
| Alpha glucosidase inhibitors | - | 90 (3.8 %) | NA |
| Thiazolidinediones | - | 22 (0.9 %) | NA |
| Others (glinides) | - | 56 (2.3 %) | NA |

**Table S1. Baseline characteristics of PARAGON-HF participants by diabetes status.** Data are presented as mean ± SD, median [Q1 – Q3] or n (%). BMI, body mass index, CCB, calcium channel blocker; CKD, chronic kidney disease; DBP, diastolic blood pressure; DPP-4, dipeptidyl peptidase-4; eGFR, estimated glomerular filtration rate; GLP-1, glucagon-like peptide-1; HF, heart failure; LVEF, left ventricular ejection fraction; MI, myocardial infarction; MRA, mineralocorticoid receptor antagonists; NT-proBNP, N-terminal prohormone B-type natriuretic peptide; SBP, systolic blood pressure; SGLT-2, sodium glucose cotransporter-2.

|  | **PARADIGM-HF** | | **PARAGON-HF** | | **Combined** | |
| --- | --- | --- | --- | --- | --- | --- |
|  | Enalapril  n=1874 | Sacubitril/valsartan  n=1904 | Valsartan  n=1184 | Sacubitril/valsartan  n=1211 | Control  n=3058 | Sacubitril/valsartan  n=3115 |
| Hypoglycemic AE, n | 44 | 53 | 31 | 51 | 75 | 104 |

**Table S2. Distribution of first investigator-reported hypoglycemic adverse events (AE) in PARAGON-HF and PARADIGM-HF participants with diabetes.**

|  | Valsartan  n=1184 | Sacubitril/valsartan  n=1211 | Adjusted difference in change from randomization (95% CI) | P value |
| --- | --- | --- | --- | --- |
| Body weight (kg) |  |  |  |  |
| Randomization | 86.5 ± 17.8 | 85.5 ± 16.9 |  |  |
| Week 48 | 87.0 ± 18.0 | 86.5 ± 17.4 | 0.29 (-0.09 to 0.67) | 0.14 |
| Week 96 | 86.6 ± 19.2 | 86.4 ± 17.5 | 0.25 (-0.33 to 0.83) | 0.40 |
| Week 144 | 86.9 ± 18.9 | 87.9 ± 17.7 | 0.77 (0.11 to 1.43) | 0.022 |
| Triglycerides (mmol/l) |  |  |  |  |
| Randomization | 1.73 ± 1.05 | 1.70 ± 0.96 |  | - |
| Week 48 | 1.83 ± 1.16 | 1.71 ± 0.96 | -0.09 (-0.16 to -0.02) | 0.012 |
| Week 96 | 1.87 ± 1.22 | 1.72 ± 0.88 | -0.14 (-0.22 to -0.07) | <0.001 |
| Week 144 | 1.85 ± 1.06 | 1.70 ± 0.92 | -0.14 (-0.23 to -0.05) | 0.003 |

**Table S3. Average body weight and triglyceride levels at randomization and at follow-up in PARAGON-HF participants with diabetes, by treatment groups.** Data shown for patients for which HbA1c values were also available at each corresponding time-point. Between-group differences in changes from randomization were adjusted for randomization values. Number of patients with body weight and HbA1c measured at randomization: n=2394 (val: n=1183, sac/val: n=1211), at week 48: n=2134 (val: n=1051, sac/val: n=1083), at week 96: n=1940 (val: n=952, sac/val: n=988), at week 144: n=1370 (val: n=690, sac/val: n=680). Number of patients with triglycerides and HbA1c measured at randomization: n=2394 (val: n=1183, sac/val: n=1211), at week 48: n=2138 (val: n=1055, sac/val: n=1083), at week 96: n=1942 (val: n=955, sac/val: n=987), at week 144: n=1370 (val: n=689, sac/val: n=681).

**Figure S2. Average body weight and triglyceride levels at randomization and at follow-up, in PARAGON-HF participants with diabetes, by treatment groups.** Data shown for patients for which HbA1c values were also available at each corresponding time-point. Error bars represent 95% confidence intervals. P from mixed models for equality of slopes.


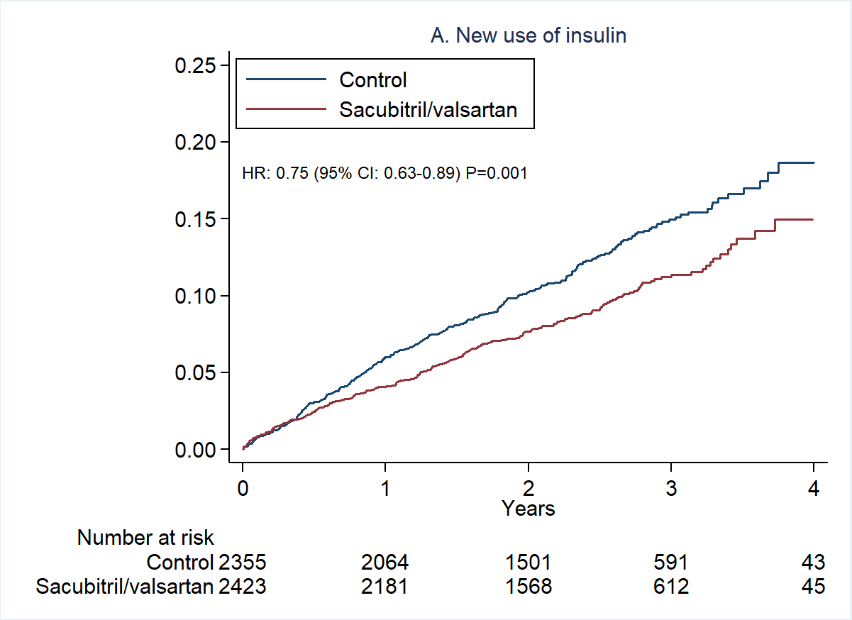


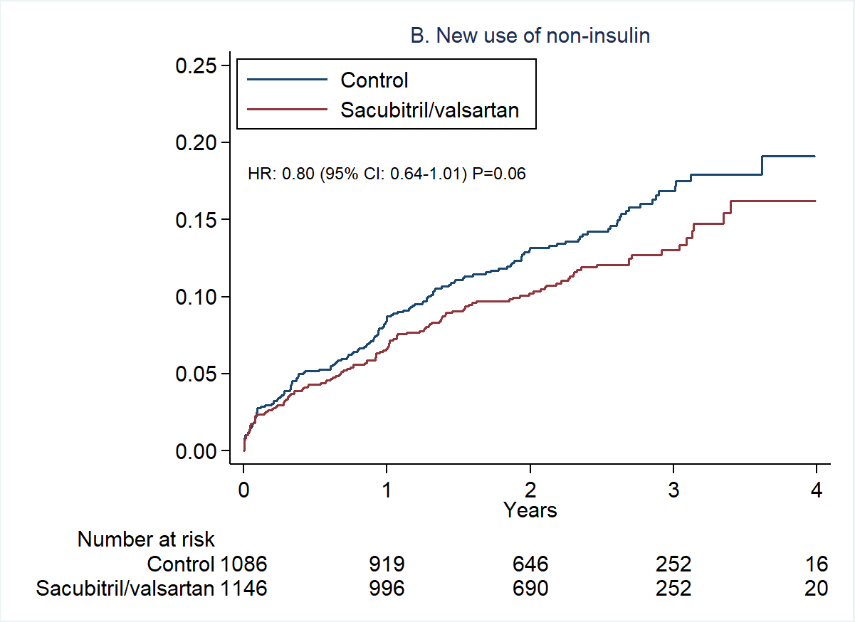


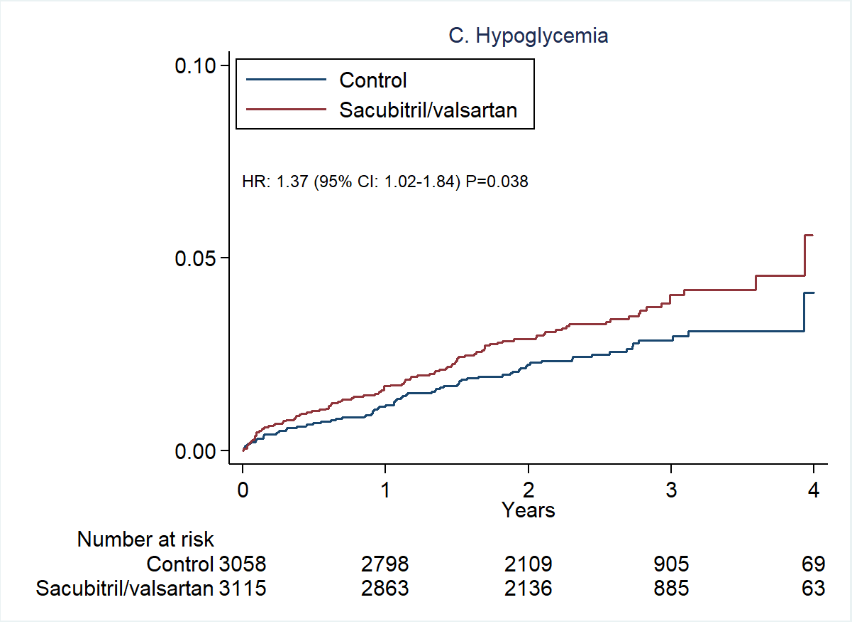


**Figure S3.** **Cumulative incidence of different outcomes in PARAGON-HF and PARADIGM-HF participants with diabetes.** (A) New use of insulin. (B) New use of non-insulin antihyperglycemic medications. (C) Hypoglycemia. HR, Hazard Ratio; CI, Confidence Interval.
